# Nuclear Shape-Shifters: Lipid and Protein Dynamics at the Nuclear Envelope

**DOI:** 10.3390/cells11244120

**Published:** 2022-12-19

**Authors:** Wolfram Antonin, Symeon Siniossoglou

**Affiliations:** 1 Institute of Biochemistry and Molecular Cell Biology, Medical School, RWTH Aachen University, 52074 Aachen, Germany; 2 Cambridge Institute for Medical Research, University of Cambridge, Cambridge CB2 0XY, UK

The nuclear envelope constitutes a selective barrier that segregates chromatin into the nucleus of eukaryotic cells. This property makes the nuclear envelope the defining morphological characteristic of the eukaryotic lineage. For many years, cell biology textbooks painted a rather inert view of the nuclear envelope as a smooth, centrally located, perinuclear circle, busy mediating the bidirectional trafficking of proteins and RNAs through nuclear pore complexes. Recent research in the field is now transforming our understanding of the nuclear envelope as a highly dynamic organelle that can undergo extensive local remodeling during physiological processes such as nuclear division or de novo nuclear pore biogenesis as well as repairing itself during cell migration or mechanical insults. The seven articles in this Special Issue tackle different aspects of how membrane lipid metabolism and the regulation of nuclear envelope protein components such as lamins and nuclear pore complexes safeguard the homeostasis of the nucleus. 

Lamins are intermediate filament proteins that coat the inner nuclear membrane and not only provide crucial mechanical support to the nucleus but also help organize the chromatin [1]. Lamin mutations in humans result in a range of pathologies, including progeria, a disorder causing accelerated ageing. Sears and Roux examine the pathways of lamin recruitment in ruptured nuclei [2]. The authors show that A-type lamins target nuclear envelope ruptures within minutes, by interacting with BAF, another protein enriched at the nuclear envelope. The observation that progeria-associated mutations in the *Lamin A* or *BAF* genes inhibit the recruitment of Lamin A protein to the ruptured sites raises the possibility that this could be a contributing factor to the disease. 

Changes in nuclear shape are associated with mutations in *lamins*, aging and many pathologies including cancer. In addition, drastic alterations in nuclear shape take place throughout the physiological life cycle of cells such as during their division or movement. Janssen et al. present an in-depth overview of image-based methods for the quantitation of nuclear morphology and nuclear envelope abnormalities [3]. Establishing these quantitative parameters is an essential tool for studies using nuclear morphology as a phenotypic read-out. 

An exciting recent finding in the field has been the identification of lipid droplets in the nucleus in a variety of cell types, although their functional significance remain enigmatic [4]. Kumanski et al. report the presence of nuclear lipid droplets in cells experiencing DNA replication stress [5]. The authors hypothesize that stalled replication forks, which are known to relocate to the nuclear envelope, may deform the inner nuclear membrane and promote lipid droplet formation at this site. 

SMPD4, a sphingomyelinase which converts sphingomyelin into ceramide and phosphocholine, has been linked to congenital microcephaly [6]. Differently to other sphingomyelinases, this lipid enzyme localizes at the endoplasmic reticulum and nuclear envelope, and it interacts with nuclear pore complex components [6,7]. Piet et al. now extend these observations by identifying additional nuclear pore complex components by BioID, most prominently Nup35, Nup155 and Aladin [8]. As Nup35 and Nup155 can deform membranes, crucial for nuclear pore complex formation, the authors speculate that SMPD4 supports this action by changing the local membrane curvature by its enzymatic action.

In a review, the same group expands on the idea that lipids and lipid metabolism at the nuclear envelope help shaping the nuclear pore membrane [9]. As some lipids have a cone or anti-cone-like shape, it is conceivable that if asymmetrically distributed along the membrane and between the lipid leaflets membranes, they shape into a concave or convex form. This could be especially important if lipid enzymes change the overall shape of lipids, such as in the case of a sphingomyelinase a cylindrical-shaped sphingomyelin, into a cone-like ceramide, and this might thus support dynamic membrane curvature changes, which could be especially relevant during nuclear pore complex formation.

In a comprehensive review, Dultz et al. provide a topical overview on the architecture of nuclear pore complexes and their life cycle [10]. The review summarizes the current knowledge of how these giant complexes are assembled into the two-membrane structure of the nuclear envelope and how these complexes are remodeled, repaired and degraded. 

Finally, Mitic et al. [11] take a closer look at Dictyostelium nuclear pore complexes by labeling individual components and following their fate through the cell cycle. The study indicates that some nuclear pore complex proteins leave this huge complex at the beginning of mitosis. Such a partial disassembly of nuclear pore complexes has been described for fungi such as Aspergilllus [12], and it is therefore important to grant spindle components access to the nuclear interior during mitosis while the nuclear envelope remains largely intact. 
